# Visual deterrents and physical barriers as non-lethal pigeon control on University of South Africa’s Muckleneuk campus

**DOI:** 10.1186/s40064-016-3559-5

**Published:** 2016-10-27

**Authors:** E. Harris, E. P. de Crom, J. Labuschagne, A. Wilson

**Affiliations:** 1 Applied Behavioural Ecology and Ecosystem Research Unit (ABEERU), Department of Agriculture and Environmental Sciences, University of South Africa, Private Bag X6, Florida, 1710 Republic of South Africa; 2 Department of Nature Conservation, Tshwane University of Technology, Private Bag X680, Pretoria, 0001 Republic of South Africa

**Keywords:** Pigeons, Non-lethal control, Eagle Eyes™, Fire (Flash) Flags, Bird spikes, Seasonality

## Abstract

A study on a population of pigeons on the University of South Africa’s Muckleneuk campus was conducted over 2 years. Counts were conducted during a baseline year (March 2013–February 2014) to establish the pigeon population index inhabiting the campus buildings, and again in the management year (August 2014–August 2015) once Eagle Eyes™, Fire (Flash) Flags, bird spikes and a combination thereof were implemented on the buildings. An efficacy reduction percentage was determined for each of the control structures. The total pigeon index on the campus declined by 50 % once the control structures were implemented. Control structures; however, differed markedly in efficacy from each other. Whilst bird spikes indicated the highest efficacy at reducing the pigeon population index, seasonality also influenced the efficacy of the control structure. Quantified understanding of the efficacy of pigeon control measures allows urban management to make informed decisions about reducing pigeon populations.

## Background

Feral pigeons were first introduced by early Europeans to Southern Africa as a free-flying domesticated species in the seventeenth century (Brooke 1981). However populations both feral (*Columba livia* Gmelin 1789) and indigenous (speckled pigeon *Columba guinea* Linnaeus 1758) have since populated urban regions throughout the subcontinent. Urban resources and lifestyles associated with human activity have enabled pigeons to establish populations as a result of the available supply and distribution of food and breeding space (Haag-Wackernagel 1995) resulting in them being considered as the most successful avian coloniser of urban spaces.

Given their long history with humans (Sossinka 1982), it is surprising that pigeons were only first considered to be problematic to the human environment in the 1930s (Sacchi et al. 2002). As pigeon populations increase people start experiencing aesthetic, vital and economic conflicts of interest (Wetherbee et al. 1964) which include the exposure to droppings and debris accumulation (Murton et al. 1972; Fitzwater 1988; Flannery 2009), public health concerns (Hutton 2005; Haag-Wackernagel and Bircher 2009), disturbance (Hutton 2005; Haag-Wackernagel and Geigenfeind 2008), structural deterioration (Hutton 2005; Giunchi et al. 2012) and to a lesser extent, bird strikes (Giunchi et al. 2012). Large flocks of pigeons have been considered to be a nuisance due to their vocalisations (Carle 1959), disturbance from squabs and breeding activities (Hutton 2005), begging (Hutton 2005), potential transmission of pathogens and parasites (Haag-Wackernagel and Moch 2004) and their sheer numbers resulting in an altered enjoyment of private and public spaces (McKeown 2008).

Pigeon control has increased substantially over the decades (Giunchi et al. 2007), with the pigeon control industry booming in the twenty-first century when public views of the birds became increasingly negative and there were calls for the systematic extermination of pigeons in urban environments (Jerolmack 2008). Subsequently, with the increase in pigeon population densities, more pest control strategies have become readily available (Giunchi et al. 2012). These control strategies have been broadly directed at either reducing pigeon numbers through increasing mortality (Haag-Wackernagel 2008; Giunchi et al. 2012), decreasing natality (Giunchi et al. 2007a, b; Haag-Wackernagel 2008; Dobeic et al. 2011) or modifying behaviour through resource management (Haag-Wackernagel 1995; Giunchi et al. 2007a, b; Haag-Wackernagel 2008). Pigeon control is often ad hoc, reactive and unsustainable (Brix et al. 2006), aimed at short-term benefit to enable continued support for pest control businesses (Murton et al. 1972).

Lethal measures have become increasingly controversial and have lost public support (Treves and Noughton-Treves 2005), while non-lethal forms of control are sustainably effective in the long term and are more acceptable to the greater public (Murton et al. 1972; Haag-Wackernagel 1984). This is particularly applicable in light of the recent listing of the feral pigeon as a Category 3 invasive species in South Africa, in terms of the Alien and Invasive Species Regulations, 2014, in terms of the National Environmental Management: Biodiversity Act of 2004 (Act 10 of 2004) of South Africa which permits the legal control of feral pigeons in urban areas (Department of Environmental Affairs 2015; SA 2015: 493).

Non-lethal pigeon control strategies are generally directed at the pigeons’ visual, auditory and tactile senses; however, habitat modification and reduction can also be achieved by physical barriers. According to Jacobs (1992), pigeons are able to see in colour and ultra-violet spectrums to aid foraging, signalling and sex recognition, and thus visual control strategies aimed at irritating or impersonating danger have varying colour spectrums. These include decoys (Harris and Davies 1998), moving lights and objects, lasers (Blackwell et al. 2002), threatening images and reflective items. Hutton and Dobson (1993) and Hutton (2005) have both found that visual deterrents have their limitations and are generally ineffective due to habituation by the pigeons.

Habitat modification through the placement of physical barriers preventing pigeons from perching on buildings and other urban structures are used widely due to their durability and acceptance by the public (Giunchi et al. 2012). Haag-Wackernagel and Geigenfeind (2008) suggest that through the restriction of entrance dimensions and the exaggeration of sloping surfaces, access prevention to ideal roosting and nesting sites can be achieved. Anti-perching devices such as sprung wires (Hutton 2005) and bird spikes (Seamans et al. 2007), or the total exclusion through netting (Hutton and Dobson 1993) can be used to deter pigeons from making use of buildings in urban environments. Cost may be a limiting factor in their implementation, and the effectiveness of these barriers can depreciate over time if these devices are not maintained (Hutton 2005).

Nevertheless, every structure and strategy has its advantages and disadvantages (Hutton and Dobson 1993). However, regardless of the control method used, if the benefits of the resources for the pigeons outweigh the costs of enduring device-related discomfort, pigeons will override any system (Haag-Wackernagel and Geigenfeind 2008). Research suggests that science seems to be lacking in quantitative reviews of various control methods and their effectiveness at reducing pigeon populations (Buijs and Van Wijnen 2001; Fukuda et al. 2008). The level of pigeon reduction of such devices needs to be quantified so that urban management can make informed decisions about the cost effectiveness and efficacy at reducing pigeon populations with regard to non-lethal control methods.

The University of South Africa’s (Unisa) Muckleneuk campus in Pretoria is host to a large number of pigeons. The birds gain access into the buildings through open access points such as loose exterior ceiling boards and open electrical and air conditioning ducts (cabling ducts) positioned on the exterior of the buildings. This easy access increases the number of protected and sheltered breeding and roosting sites available. It has also increased health concerns relating to the build-up of their faeces and associated fungi, nest mites and bird lice which have been reported to infest the offices and affect the staff working in certain buildings on campus. Faeces and accumulated nesting material build-up on the various external structures of the campus buildings have become an issue of concern. According to Ntshoe (pers. comm. 2013), large financial investments have been made in order to manage the birds and their associated problems on an ad hoc and reactive basis.

This paper evaluates non-lethal humane pigeon control strategies with particular focus on visual deterrents and physical barriers on the Unisa Muckleneuk campus and will examine the following objectives and null hypothesis.

## Objectives

- To determine if the control structures have decreased the pigeon population index on campus.
- To determine if pigeons will move from a building with control structures to an untreated building.
- To establish if seasonality influences the efficacy of control structures.
- To validate the industry percentage reduction claims with regard to control structures.
- To evaluate the efficacy percentage reduction of Eagle Eyes™, Fire Flags, bird spikes and a combination thereof on the pigeon population index.

## Null hypothesis

- Control structures, namely Eagle Eyes™, Fire Flags, bird spikes and combinations of these, will not significantly differ from each other in efficacy at reducing the pigeon population index.

## Study area

Unisa (−25.76776, 28.199158) is situated on top of a hill (1411.19 m above sea level) near the central business district of Pretoria in Gauteng in South Africa. The city is surrounded by the Magaliesburg mountain range in the transitional zone between the Central Bushveld and Moist Highveld Grassland vegetation types (Kruger 2004). The city has a moderate, warm temperate climate with an annual minimum and maximum temperature average of 13 °C (June) and 24 °C (January) respectively which was measured during the course of the study. According to the South African Weather Service (2010), precipitation averages 677 mm, while relative humidity ranges between 44 and 75 % annually. Pretoria experiences 3 254 h of sunshine a year with an average of 2.4–2.7 days of cloud cover recorded annually (South African Weather Service 2010). The Pretoria region within a 20 km radius of Unisa includes commercial, industrial, suburban and rural areas, with farming and crop (maize, soya, sorghum and sunflowers) production in the surrounding districts (Collett 2015).

The campus is located within a green belt which includes the surrounding Groenkloof Nature Reserve, Fountains Valley, Apies River, Voortrekker Monument and Freedom Park. Various small mammals and bird species inhabit the university’s grounds. These include avian migrants and small raptors.

**Fig. 1:**
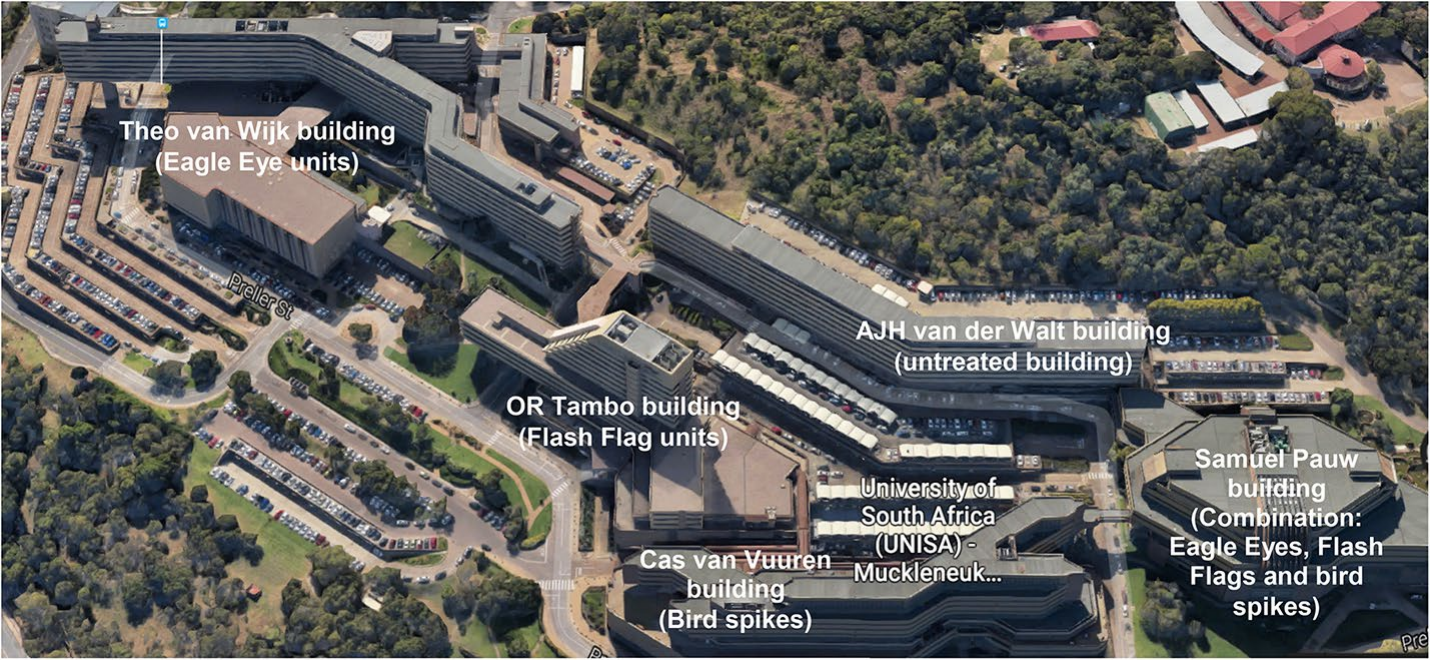
The University of South Africa’s Muckleneuk campus in Pretoria, indicating the five buildings and their respective pigeon control structures, in proximity to each other (GoogleMaps 2016)

Established in 1972, the Muckleneuk campus consists of seven administrative and academic buildings; however, for the purpose of this study only the following five of the seven buildings were investigated as part of the pigeon research: Theo van Wijk building, OR Tambo building, AJH van der Walt building, Cas van Vuuren building and Samuel Pauw building (Fig. 1). Each building is unique in its design, providing various roosting and nesting site possibilities for the pigeon population index on the campus. Academic and administrative offices are positioned lengthwise along the buildings and face out onto balconies.

Theo van Wijk, the largest building positioned on the far western side of the campus, has 11 levels uniform in design with balconies and exterior cabling ducts running the length of the building. Due to its y-shape, the building offers two north facing and two south facing aspects. The Philadelphia cafeteria is positioned on the third floor, which includes an extensive catering balcony.

The AJH van der Walt building is positioned on the northern side of the campus facing an undeveloped vegetated mound which meets the campus’ northern boundary. All seven levels are continuous in balcony and cabling ducts design.

To the east of the campus is the library, housed in the Samuel Pauw building, roughly hexagonal in shape with eight levels and continual balconies. Beyond this building towards the campus boundary in the east is parking space and natural vegetation.

OR Tambo, the administrative building, is positioned to the south. It is the tallest building on campus with 14 levels. Balconies and cabling ducts provide uniform exterior structural design, with the exception of the Good Hope cafeteria and balcony positioned on level four.

Adjacent to the OR Tambo building is the Cas van Vuuren building with seven levels and no exterior cabling ducts positioned above its balconies. Natural areas extend to the southern and south-western boundaries.

A characteristic of all the buildings are the loose, broken or open exterior ceiling boards and cabling ducts which provide additional roosting and breeding space for the pigeon population index on campus.

## Methods

This study took place over two years. During the first year data was collected for a full year from the beginning of March 2013 to the end of February 2014 to provide a baseline year to determine the index of the pigeon population inhabiting the buildings on the Muckleneuk campus. This data was used to determine the efficacy of the control measures implemented on the campus buildings during the second year (August 2014–August 2015).

For each year adult and juvenile pigeons were counted during the pigeons’ bimodal foraging activity periods, which have been recorded to peak in the morning and afternoon (Rose et al. 2006; Soldatini et al. 2006). These counts took place early morning during the first 2 h after sunrise and again in the evening during the last 2 h before sunset, once a week for 52 weeks. If the particular chosen day for counting experienced extreme weather conditions, then the next consecutive day with fine weather was chosen and documented.

The observer maintained a standard designated route in a west to east direction, counting each of the campus’ five buildings during the course of the research period. Observations were aided binoculars, digital camera and dictaphone, later transcribed onto data sheets. Double counts of individuals taking off and perching on the same building was taken into consideration and avoided. As the pigeons were wild and free roaming, the exact number of pigeons on campus could not be determined. An increase or decrease in the number of pigeons counted was in essence a reflection of the unknown population size and directly correlated to an increase or decrease in pigeon presence on campus (Gregory et al. 2005). Presence was represented as an index to monitor the extent of the increases or decreases as actual numbers could not be attained through the methodology implemented. As the index reflects a portion of the pigeon population, a portion which may be change over time, methodology was therefore standardised to mitigate variability (Johnson 2008). The paper will therefore refer to indices to convey the extent of the pigeon presence, and its changes over time. The results of the baseline year were therefore interpreted as an index of pigeon population size. The use of the term ‘population’ in this study does not refer to a biological population as a demographic unit but rather as a population index indicative of the census technique employed.

During the second year, once the baseline year was completed, various pigeon control structures were installed on four buildings (Theo van Wijk building, OR Tambo building, Cas van Vuuren building and Samuel Pauw building) for the management year (August 2014–August 2015, 52 weeks). One of the buildings, AJH van der Walt building, was used as a control building without any pigeon control structures or strategies to determine whether pigeons deterred from surrounding buildings with control structures simply moved to an untreated building as suggested by Mooallem (2006).

**Fig. 2:**
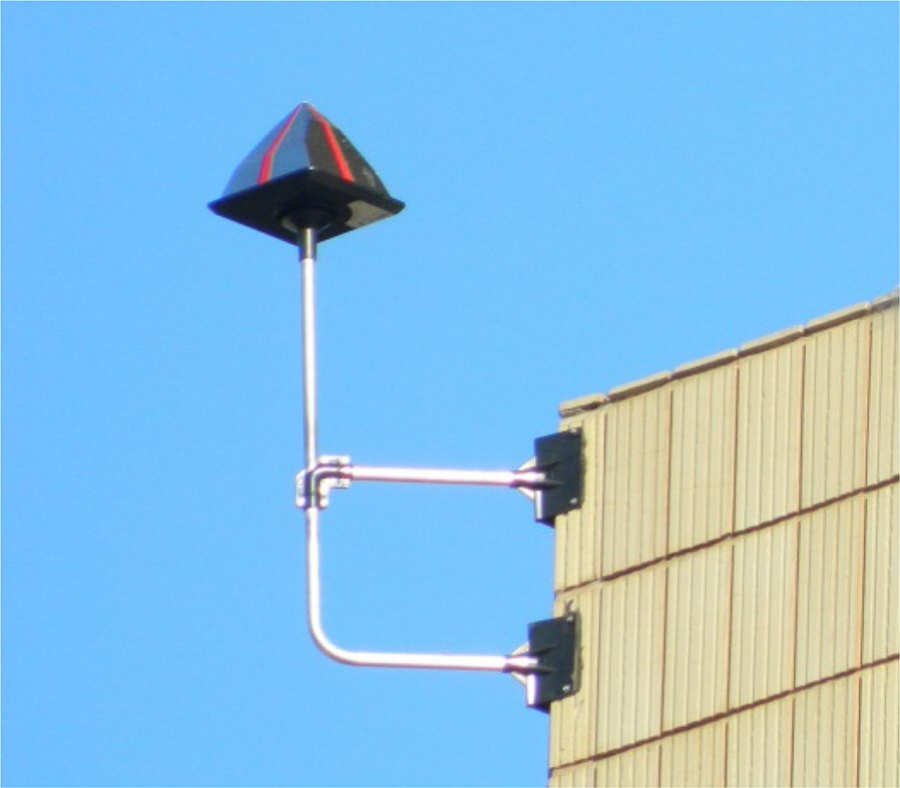
Eagle Eye™ unit evaluated as a pigeon deterrent on the University of South Africa’s Muckleneuk campus

**Fig. 3:**
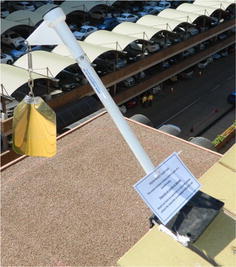
Flash Flag unit evaluated as a pigeon deterrent on the University of South Africa’s Muckleneuk campus

**Fig. 4:**
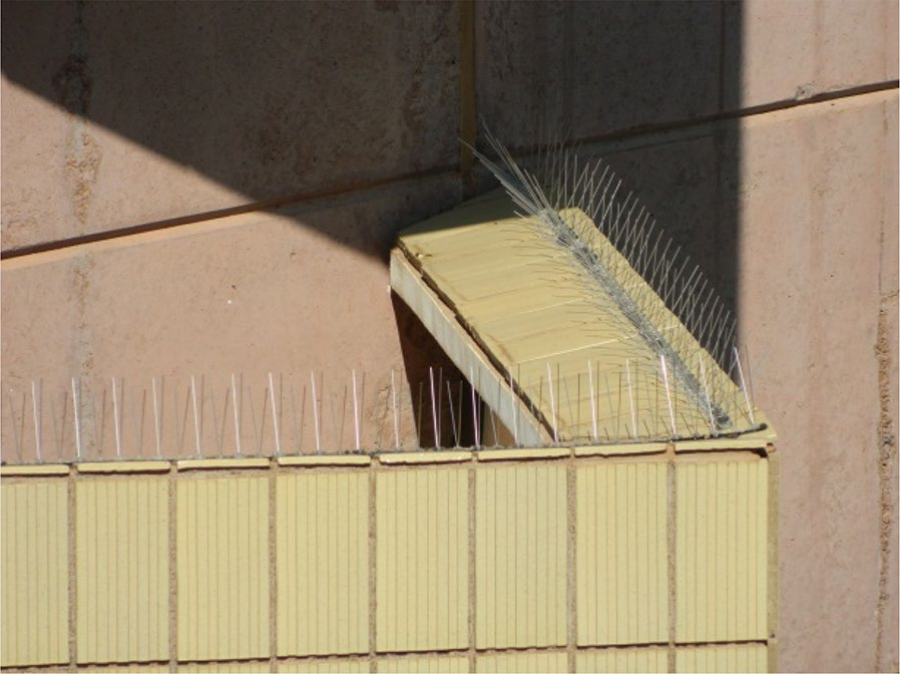
Bird spikes evaluated as a pigeon deterrent on the University of South Africa’s Muckleneuk campus

Pigeon control structures chosen for this study included Eagle Eyes™ (visual deterrent) which are rotating prisms that reflect light within the ultra violet spectrum designed to interfere with the pigeons’ line of flight as the light causes a distraction (Eagle Eye 2015) (Fig. 2); Fire (Flash) Flags (visual deterrent), made from reflective gold and silver plastic, are designed to move with the wind to give the impression of fire and danger (Eagle Eye 2015) (Fig. 3); bird spikes (physical barrier), which are dual-pronged, stainless steel spikes continuously placed along the ledge of a building aimed at preventing pigeons from perching (Fig. 4); and the combination of the above mentioned control structures (Eagle Eyes™, Fire Flags and bird spikes) recommended by a well-known pest control company in South Africa for optimal pigeon deterrence.

The pest control company marketing and selling the pigeon control structures identified the optimal placement of each control structure tested in this study per building on campus to ensure that each building was suitably covered by the chosen structure for pigeon control purposes.

The largest building, Theo van Wijk building, is positioned on the far western side of the campus. Due to its extensive size and y-shape creating two north and two south facing aspects, Eagle Eye™ units were chosen. Sunlight reflected by the 36 units was able to cover a greater surface area relative to the other control structures, and its proximity to other buildings contributed to the control structure choice as light from the units would affect surrounding buildings thus influencing their respective control strategies. Units were placed on each balcony of the 11 stories and at regular intervals along the roof (north and south facing).

OR Tambo, the tallest building with 14 levels, was selected to test the Fire Flags due to the updraft of wind that is experienced at such high altitudes. Eighty units were placed along the levels (north and south facing).

The Cas van Vuuren building was identified for use of bird spikes as the building does not have the exterior electrical and air conditioning ducts (cabling ducts) which are positioned just below the balcony ceilings above the office windows of other buildings on campus. These ducts provide ideal sites for pigeons to roost and nest on. A single continuous strand of bird spikes (1720 m) was positioned along the length of the balcony ledge on all seven levels (north and south facing).

The university’s library, Samuel Pauw, hexagonal in shape, was chosen for the implementation of the control structure combination recommended by the pest control company. This included six Eagle eye™ units, 12 Fire Flag units and 2790 m of bird spikes applied to the eight levels of the building.

The same methodology used in year one was applied in the second year to determine the efficacy of the control structures on the pigeon population index. Arithmetic means and standard errors of the monthly pigeon population indices are depicted graphically over the course of the two years. Pigeon population index and efficacy rate was determined by calculating the percentage change in the number of counts of pigeons between the baseline year and management year in which the control structures were implemented. This indicated the reduction in percentage of each control structure on the pigeon population index.

To test whether or not there was a difference in the mean efficacy percentages between the different control structures a one-way ANOVA was used. Where significant differences between the control structures were observed, Bonferroni post hoc tests were employed to determine which of the control structures differed significantly from each other in one-to-one comparisons.

Institutional ethical clearance and permission (2013/CAES/017) was received for the research.

## Results

**Fig. 5:**
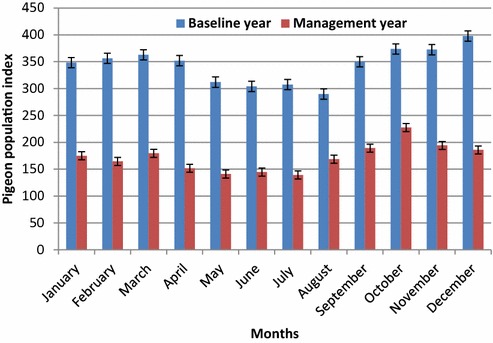
Pigeon population index between the baseline year (March 2013–February 2014) and the management year (August 2014–August 2015) indicating standard error

The mean pigeon population index declined by 50 % between the baseline study year (March 2013–February 2014) (x = 344 individuals; SE = 10) and the management year (August 2014–August 2015) (x = 172 individuals; SE = 7) once the control structures were installed on the buildings (Fig. 5).

As a natural decline in the campus’ pigeon population on AJH van der Walt (untreated building) between the baseline (year 1) and management year (year 2) was observed. The control structure efficacies values were weighted proportionally to this decline in the population index.

Fire Flags reduced the pigeon population index by an average of 33 %, while Eagle Eyes™ indicated a mean reduction in the pigeon population index by nearly 40 %. The combination of control structures resulted in a mean reduction of 45 %, while bird spikes reduced the pigeon population index the most by a mean of nearly 70 %.

**Fig. 6:**
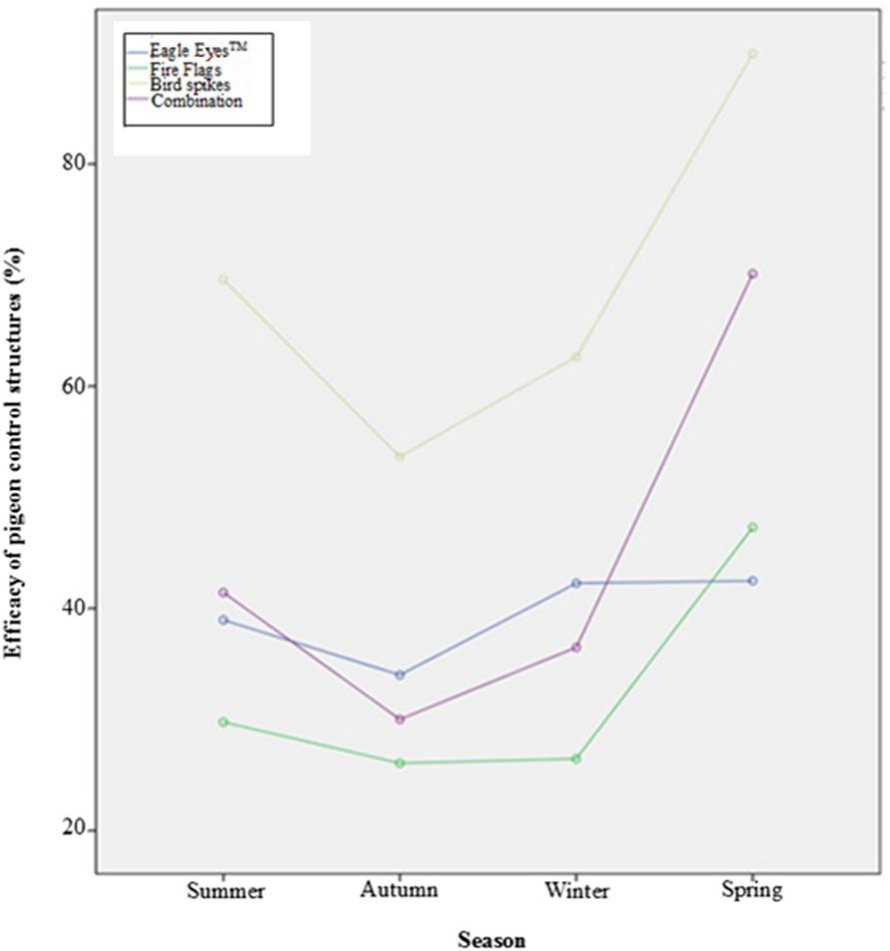
The efficacy of control structures over the seasons during the management year on the Muckleneuk campus (August 2014–August 2015)

 The efficacy of control structures on the pigeon population index increased in the warmer seasons, thus structures were more efficient at reducing the pigeon population index in spring than in autumn. While Fire Flags were the least efficient, bird spikes were the most effective at reducing the pigeon population index on the Muckleneuk campus across all seasons (Fig. 6).

As the *p* value was very small, there was a significant difference in mean efficacy value between the different control structures (F = 5.666, *p* < .001). Confidence intervals (95 %) were included to incorporate the standard errors into the results.

The mean value of efficacy did not differ significantly between Eagle Eyes™, Fire Flags *p* = .144, 95 % CI [−1.20, 15.28]; and the combination *p* = .646, 95 % CI [−13.24, 3.24], but did significantly differ from bird spikes *p* = .000, 95 % CI [−37.74, −21.26] (Table 1).

**Table 1:** Bonferroni post hoc test indicating one-to-one comparisons of the efficacy values of each control structure at reducing the pigeon population index during the management year on the Muckleneuk campus (August 2014–August 2015)

Pigeon control strategy	Pigeon control strategy	Significance at 95 % confidence
Eagle Eyes™	Fire Flags	.144
Eagle Eyes™	Bird spikes	.000
Eagle Eyes™	Combination	.646
Fire Flags	Bird spikes	.000
Fire Flags	Combination	.001
Bird spikes	Combination	.000

The mean value of efficacy did not differ significantly between Fire Flags and Eagle Eyes™ *p* = .144, 95 % CI [−15.28, 1.20]. However, the efficacy of Fire Flags did significantly differ between bird spikes *p* = .000, 95 % CI [−44.78, −28.30] and the combination *p* = .001, 95 % CI [−20.28, −3.80] (Table 1).

The mean value of efficacy did significantly differ between bird spikes and the other control structures, namely Eagle Eyes™ *p* = .000, 95 % CI [21.26, 37.74], Fire Flags *p* = .000, 95 % CI [28.30, 44.78], and the combination *p* = .000, 95 % CI [16.26, 32.74] (Table 1).

The mean value of efficacy did not differ significantly between the combination and Eagle Eyes™ *p* = .646, 95 % CI [−3.24, 13.24], but did significantly differ from Fire Flags *p* = .001, 95 % CI [3.80, 20.28] and bird spikes *p* = .000, 95 % CI [−32.74, −16.26] (Table 1).

## Discussion

Knowledge of population processes and parameters, activity patterns, abundance, life requirements and resource use of pigeons (Fitzwater 1970; Godin 1994; Johnson 2000) influences the choice and placement of control structures as well as their efficacy at reducing the population (Seamans et al. 2013). Furthermore, numerous studies (Seamans et al. 2003; Dinetti 2006; Giunchi et al. 2012) have noted the positive benefits of integrated pest management, rather than a single method of control (Shea et al. 2000). Control methods focused on multiple scare devices such as Eagle Eyes™ and Fire Flags combined with habitat modification (Booth 1994) and limiting ecological resources (Giunchi et al. 2012) appear to result in the successful reduction of pigeon populations (Seamans et al. 2003). This was found to be true on the Muckleneuk campus as the pigeon population index declined by 50 % as a result of the various control structures placed on the campus buildings. However, few studies have quantified the efficacy that different control structures contribute to the overall decline in a pigeon population index. Instead control structures are recommended based upon informal reviews and incidental observations (Seamans et al. 2007; Fukuda et al. 2008), whereas our study found the different control structures to be significantly different in efficacy.

The efficacy of bird spikes to reduce the pigeon population index on campus was significantly different from the other control methods, and thus the null hypothesis was rejected. Bird spikes were found to be the most successful in reducing the pigeon population (nearly 70 %) as pigeons were physically hindered from perching on the treated building. Seamans et al. (2007) described similar findings at an airport; their research found the anti-perching spikes to be effective against preventing pigeons from perching on buildings. Based on biological principles, the spikes deny access to sites selected by pigeons (Harris and Davies 1998). Nonetheless faeces and debris caught in the spikes can render them redundant and ineffective (Barnes 1997) when pigeons build their nests on top of this accumulation. Ongoing maintenance and monitoring is therefore essential to retain efficacy.

Scare devices such as Fire Flags and Eagle Eyes™ are considered to have limited efficacy at reducing pigeon populations (Harris and Davies 1998; Fukuda et al. 2008). This was found to be the case with the units placed on the Muckleneuk campus. There is much literature that describes habituation to the units as a limiting factor of visual deterrents (Godin 1994; Harris and Davies 1998). Due to the interdisciplinary nature of this study, the human component of staff on campus influenced the efficacy of the Eagle Eyes™ and Fire Flags as a number of units were removed and vandalised during the course of this study thus resulting in less than optimal unit placement and quantity.

Research conducted on structures with similar deterrent components to Fire Flags such as reflecting tape (Bruggers et al. 1986; Harris and Davies 1998), mylar ribbon (Tobin et al. 1988) and metallic streamers (Christensen 1996) describes similar findings of their inefficiency at deterring bird species. Furthermore, Harris and Davies (1998) point out the lack of biological basis regarding the Fire Flags and the limited application in deterring birds from areas. In fact, Fire Flags were found to be the least efficient control structure (33 %), and did significantly differ from bird spikes and the combination.

Eagle Eye™ units were also found to have limited efficacy at reducing the pigeon population index on campus, and differed significantly from the bird spikes. Research conducted on a similar European device, Peaceful Pyramid^®^, supported these findings as the reflecting mirrors were only marginally effective at altering pigeon behaviour (Seamans et al. 2003; Fukuda et al. 2008). According to the company’s literature, the Eagle Eye™ unit has been successfully used to deter various avian species, including pigeons (Eagle Eye 2015), but the efficacy of Eagle Eye™ units has thus far been based on subjective estimates and anecdotal reports. The limited efficacy can be attributed to habituation to the units (Fukuda et al. 2008; Giunchi et al. 2012). In other avian studies, flashing lights and mirrors, the fundamental control attributes of the Eagle Eye™ units, were also found to be ineffective (Seamans et al. 2001) at deterring birds in urban environments.

The pest control industry recommends a combination of Eagle Eyes™, Fire Flags and bird spikes in order to achieve maximum efficacy at reducing pigeon populations. According to the literature supplied by Eagle Eye (2015), a reduction of up to 80 % of pigeon populations can be expected. In spite of this, the combination applied to the Samuel Pauw building was less successful than the spikes-only application on another building, but significantly different from Eagle Eye™ and Fire Flag units. This is in contrast to the assumption that the combination would be more effective than its individual parts. However, it should be mentioned here that the building on which the combination was applied had numerous open exterior cabling ducts and open or loose exterior ceiling boards. These provided the pigeons with alternative sites to the spikes which reduced the perching surface area of the balcony ledges, consequently limiting the effect of the combination on the pigeon population index. According to Jerolmack (2008), the life-sustaining processes of pigeons are often ignored when control measures are implemented. The combination of methods used on the Samuel Pauw building supports this view, as the carrying capacity of the pigeon population index was not optimally reduced for this building due to the availability of alternative untreated space on the building. The combination did, however, differ significantly from the bird spikes and Fire Flags.

Further replications of the pigeon control measures on numerous buildings would have improved the possibility of repeated results, however due to building access and financial implications; this study was limited to building availability.

On a larger scale, pigeon control does not influence the actual population size (Krimowa 2012) but simply displaces individuals away from the deterring systems to untreated buildings or sites (Mooallem 2006; Geigenfeind 2013). Nevertheless, a decline in the pigeon population index on campus after the control structures were implemented was noted and a 23 % reduction in the pigeon population index between the baseline year and the management year on the untreated building occurred. It can therefore be inferred that no pigeons which previously inhabited the treated buildings moved to the untreated control building.

Another contributing factor which affects the effectiveness of avian deterrents is seasonality (Seamans et al. 2013). Climatic conditions, environmental changes and food availability all have an influence on the behaviour of pigeons and their subsequent tolerance of control structures. The efficacy of structures was found to increase during the warmer months corresponding with the natural pigeon population index fluctuation on campus (spring (${\bar{\text{x}}}$ = 365 pigeons; SE = 4) and summer (${\bar{\text{x}}}$ = 367 pigeons; SE = 8) seasons in comparison to the autumn (${\bar{\text{x}}}$ = 342 pigeons; SE = 8) and winter (${\bar{\text{x}}}$ = 300 pigeons; SE = 3) seasons) (Harris et al. 2016). Pigeons on the Muckleneuk campus which breed year-round with a peak between July and October (Harris et al. 2016), indicated a higher tenacity for tolerating the control structures in spring. According to Curio and Regelmann (1983), there is a trade-off between conflicting demands in great tits, and this study too found that pigeons on campus were willing to endure the discomfort of deterrents in order to rear their young. Wildlife regularly makes decisions that are crucial to their survival and fitness (Conradt and Roper 2005). This was evident with the pigeons on the Muckleneuk campus as the population index on the campus buildings were found to be inversely related to the availability of their main food source, agricultural crops (Harris et al. 2016). Pigeons on campus rather directed their energy into foraging locally than travelling to surrounding agricultural areas in spring due to the limited crop availability (Harris et al. 2016). As a result more pigeons were visible on the buildings. The higher spring population index in the visual deterrents may also imply that the structures installed may ultimately not impact on pigeons’ behaviour in a significant way.

Even though Eagle Eyes™, Fire Flags and the combination of control structures presented an irritant to the pigeons inhabiting the buildings, pigeons were willing to tolerate the discomfort. As a result the seasonal efficacy of these measures was low. This is in contrast to the bird spikes installed on the Cas van Vuuren building which did not provide any additional perching space in the form of exterior cabling or open ceiling boards. Pigeons were physically unable to perch on the balcony ledge where bird spikes had been placed, which resulted in a high seasonal efficacy at reducing the pigeon population index.

All control structures on the campus buildings were found to be the least efficient during the autumn months. According to Pulliam (1976) and McCleery (1978), different behavioural options of wildlife result in a continual shift in relative costs and benefits. Due to the colder temperatures, pigeons were hidden as a result of thermal factors (Harris et al. 2016) and consequentially also from the observer. Autumn also coincides with a biologically important season in the pigeon life cycle, namely moulting. As costly energy is diverted for feather regrowth and plumage change (Murton 1966; Johnston and Janiga 1995), pigeons are relatively inactive as opposed to the rest of the year in order to conserve energy.

## Conclusion

The control of pigeons has become increasingly humane and non-lethal, with consideration for ecological processes and sustainability. Single methods of control are no longer viable nor sustainable, and successful management of pigeons can only be achieved with integrative measures as seen on the Muckleneuk campus.

This study found visual scare devices to be far less effective at reducing the pigeon population index on campus than the physical exclusion and habitat modification of the bird spikes. However, as site-specific environmental factors and ecological resource availability affect pigeon control, this is not to say that Fire Flags and Eagle Eyes™ will not be effective on a different site or building. They were simply ineffective on the Muckleneuk campus. A further comparative study evaluating the effect of control structures recommended by the pest control industry should be undertaken independently without the influence of people.

Similarly the combination of control structures targeted at maximising pigeon reduction would improve in efficacy if the open cabling ducts and ceiling boards that provide alternative perching sites are attended to. According to Ryzhov and Mursejev (2010), the success and efficacy of control structures to reduce pigeon populations depend on the conditions of usage. The success of control structures is therefore dependent on the context of application and factors influencing a site. Blanket statements on the expected percentage reduction of pigeon populations by control structures cannot be guaranteed as each site and pigeon population index interaction is unique as seen on the Muckleneuk campus. Similar studies at other sites considering building design and optimal pigeon control structure placement would need to be undertaken to confirm a range of efficacies for visual deterrents and physical barriers specific for managing pigeons.

Though seasonality affects the efficacy of control structures, with spring and autumn indicating respective peaks and dips, pigeons did not move from the treated to the untreated buildings as previously thought. This does not necessarily imply that pigeons did not move between treated buildings to limit their exposure to the deterrent, but merely that this study did not investigate this aspect.

As urbanisation continues to expand, human-wildlife conflicts involving pigeons are expected to increase, and subsequently an increase in the demand for control. The effect of control structures at reducing pigeon populations has not yet been extensively quantified (Seamans et al. 2007; Fukuda et al. 2008). This is a vital component of urban management in order to be able to make informed decisions about the cost effectiveness and efficacy at reducing pigeon populations relating to non-lethal control methods. Integrative pest management, including a combination of measures based on sound biological principles (Davis 1974), combined with time and use variation will result in more sustainable pigeon population reductions in urban environments.

There is a need for greater scientific understanding of the efficacy of non-lethal pigeon control measures in order to be able to manage pigeon populations to ensure healthy, socially acceptable standards (Dobeic et al. 2011).

## References

[CR2] Barnes TG (1997) Managing urban pest bird problems in Kentucky. University of Kentucky Cooperative Extension Service

[CR3] Blackwell BF, Bernhardt GE, Dolbeer RA (2002). Lasers as nonlethal avian repellents. J Wildl Manag.

[CR4] Booth TW (1994) Bird dispersal techniques. In: Hygnstrom SE, Timm RM, Larson GE (eds) Prevention and control of wildlife damage. University of Nebraska-Lincoln, p 58

[CR5] Brix A, Brydon T, Davidian E, Dinse K, Vidyarthi S (2006) Toward sustainable campus communities: evaluating alternative development scenarios. Dissertation, University of Michigan

[CR6] Brooke RK (1981). The feral pigeon: a ‘new’ bird for the South African list. Bokmakierie.

[CR7] Bruggers RL, Brooks JE, Dolbeer RA, Woronecki PP, Pandit RK, Tarimo T, Hoque M (1986). Responses of pest birds to reflecting tape in agriculture. Wildl Soc Bull.

[CR8] Buijs JA, Van Wijnen JH (2001). Survey of feral rock doves (*Columba livia*) in Amsterdam, a bird-human association. Urban Ecosyst.

[CR9] Carle R (1959). Die Vorbereitung zur Bestandsregelung unter den verwilderten Haustauben in den Städten. Städtehygiene.

[CR10] Christensen W (1996). Creative pigeon management. New Hamps Audubon.

[CR11] Collett A (AnnelizaC@daff.gov.za) (2015) Agricultural crop list. [Email to:] Harris, E. (emmaharris09@gmail.com) Oct 21

[CR12] Conradt L, Roper TJ (2005). Consensus decision making in animals. Trends Ecol Evol.

[CR13] Curio EK, Regelmann K (1983). An anti-predator response in the great tit (*Parus major*): is it tuned to predator risk?. Oecologia.

[CR14] Davis PJ (1974). Fundamentals of bird scaring: a laboratory approach. Ann Appl Biol.

[CR68] Department of Environmental Affairs see South Africa. Department of Environmental Affairs

[CR15] Dinetti M (2006). Urban avifauna: is it possible to live together?. Vet Res Commun.

[CR16] Dobeic M, Pintarič S, Vlahović K, Dovč A (2011). Feral pigeon (*Columba livia*) population management in Ljubljana. Vet Arhiv.

[CR1] Eagle Eye Database. http://www.eagleeye.co.za/?gclid=CLqbuaXd8ckCFWXnwgod0yoLwQ. Accessed 10 Sept 2015

[CR17] Fitzwater WD (1970) Sonic systems for controlling bird predations. In: Proceedings of the Bird Control Seminars, p 203

[CR18] Fitzwater WD (1988) Solutions to urban bird problems. In: Proceedings of the thirteenth vertebrate pest conference, pp 254–259

[CR19] Flannery MC (2009). The value of pigeons. Am Biol Teach.

[CR20] Fukuda Y, Frampton CM, Hickling GJ (2008). Evaluation of two visual birdscarers, the Peaceful Pyramid^®^ and an eye-spot balloon, in two vineyards. N Z J Zool.

[CR21] Geigenfeind I (2013) On the biology and epidemiology of the feral pigeon (*Columba livia*). Thesis, University of Basel

[CR22] Giunchi D, Gaggini V, Baldaccini NE (2007). Distance sampling as an effective method for monitoring feral pigeon (*Columba livia* f. *domestica*) urban populations. Urban Ecosyst.

[CR23] Giunchi D, Baldaccini NE, Sbragia G, Soldatini C (2007). On the use of pharmacological sterilisation to control feral pigeon populations. Wildl Res.

[CR24] Giunchi D, Albores-Barajas YV, Baldaccini NE, Vanni L, Soldatini C (2012) Feral pigeons: problems, dynamics and control methods. In: Soloneski S (ed) Integrated pest management and pest control—current and future tactics. Intech

[CR25] Godin AJ (1994) Birds at airports. In: Hygnstrom SE, Timm RM, Larson GE (eds) Prevention and control of wildlife damage. University of Nebraska-Lincoln, p 56

[CR27] Gregory RD, Gibbons DW, Donald PF, Sutherland WJ, Newton I, Green RE (2005). Bird census and survey techniques. Bird ecology and conservation: a handbook of techniques.

[CR28] Haag-Wackernagel D (1984) Ein Beitrag zur Ӧkologie der Stadttaube. Thesis, Universitӓt Basel

[CR29] Haag-Wackernagel D (1995). Regulation of the street pigeon in Basel. Wildl Soc Bull.

[CR30] Haag-Wackernagel D (2008) Feral pigeon management. Available via DIALOG. www.Anatomie.unibas.ch/integrativeBiology/hag/Feral-Pigeon/Strassentauben/pigeon-management.html. Accessed 12 Feb 2013

[CR31] Haag-Wackernagel D, Bircher AJ (2009). Ectoparasites from feral pigeons affecting humans. Dermatol.

[CR32] Haag-Wackernagel D, Geigenfeind I (2008). Protecting buildings against feral pigeons. Eur J Wildl Res.

[CR33] Haag-Wackernagel D, Moch H (2004). Health hazards posed by feral pigeons. J Infect.

[CR34] Harris RE, Davies RA (1998). Evaluation of the efficacy of products and techniques for airport bird control.

[CR35] Harris E, de Crom EP, Labuschagne J, Wilson A (2016). Urban environment use by speckled (*Columba guinea)* and feral (*Columba livia)* pigeons on the University of South Africa’s Muckleneuk campus. Appl Ecol Environ Res.

[CR36] Hutton R (2005) Method statement for the control of feral pigeons. Available via DIALOG. http://www.handr.co.uk/literature/feral_pigeons.htm. Accessed 5 Dec 2012

[CR37] Hutton TC, Dobson J (1993). The control of feral pigeons: an independent approach. Struct Surv.

[CR38] Jacobs GH (1992). Ultraviolet vision in vertebrates. Am Zool.

[CR39] Jerolmack C (2008). How pigeons became rats: the cultural-spatial logic of problem animals. Soc Probl.

[CR40] Johnson RJ (2000) Management of pest birds in urban environments. In: Proceedings urban pest conference, Lincoln, NE, 19–20 January

[CR41] Johnson DH (2008). In defence of indices: the case of bird surveys. J Wildl Manag.

[CR42] Johnston RF, Janiga M (1995). Feral pigeons.

[CR43] Krimowa S (2012) Pigeons and people: resource ecology and human dimensions of urban wildlife. Dissertation, University of Wellington

[CR44] Kruger AC (2004) Climate of South Africa. Climate Regions. WS45. South African Weather Service, Pretoria, South Africa

[CR45] Mccleery RH, Krebs JR, Davies NB (1978). Optimal behaviour sequences and decision making. Behavioural ecology: an evolutionary approach.

[CR46] Mckeown D (2008) Toronto Pigeon feeding. Internal Document

[CR47] Mooallem J (2006) Pigeon wars. Available via DIALOG. http://www.nytimes.com/2006/10/15/magazine/15pigeons.html. Accessed 12 Feb 2015

[CR48] Murton RK (1966). Natural selection and the breeding seasons of the stock dove and wood pigeon. Bird Study.

[CR49] Murton RK, Thearle RJP, Thompson RJP (1972). Ecological studies of the feral pigeon *Columba livia var. l*. Population, breeding biology and methods of control. J Appl Ecol.

[CR50] Ntshoe L (2013) Cleaning manager, University Estates, UNISA. Personal Communication

[CR51] Pulliam HR, Bateson PPG, Klopfer PH (1976). The principle of optimal behaviour and the theory of communities. Perspectives in ethology.

[CR52] Rose E, Nagel P, Haag-Wackernagel D (2006). Spatio-temporal use of the urban habitat by feral pigeons (*Columba livia*). Behav Ecol Sociobiol.

[CR53] Ryzhov SK, Mursejev MR (2010). Trained goshawks against pigeons. J Raptor Conserv.

[CR69] SA see SOUTH AFRICA

[CR54] Sacchi R, Gentilli A, Razzetti E, Barberi F (2002). Effects of building features on density and flock distribution of feral pigeons *Columba livia var.domestica* in an urban environment. Can J Zool.

[CR55] Seamans TW, Lovell CD, Dolbeer RA, Cepek JD (2001) Evaluation of mirrors to deter nesting Starlings. USDA National Wildlife Research Centre, 613

[CR57] Seamans TW, Barras SC, Patton ZJ (2003) Are birds scared by rotating mirrors? In: Proceedings of the bird strike committee USA Canada 5th annual meeting, Ontario, Toronto, 18–21 Aug 2003

[CR56] Seamans TW, Barras SC, Bernhardt GE (2007). Evaluation of two perch deterrents for starlings, blackbirds and pigeons. Int J Pest Manag.

[CR58] Seamans TW, Martin JA, Belant JL (2013) Tactile and auditory repellents to reduce wildlife hazards to aircraft. In: Wildlife in airport environments. USDA National Wildlife Research Center - Staff Publications. Paper 1650

[CR59] Shea K, Thrall PH, Burdon JJ (2000). An integrated approach to management in epidemiology and pest control. Ecol Lett.

[CR60] Soldatini C, Mainardi D, Baldaccini NE, Giunchi D (2006). A temporal analysis of the foraging flights of feral pigeons (*Columba livia* f. *domestica*) from three Italian cities. Ital. J Zool.

[CR61] Sossinka K (1982). Domestication in birds. J Avian Biol.

[CR62] South Africa (2015) National Environmental Management: Biodiversity Act 2004 (Act No, 10 of 2004) Draft Amendments to the Alien and Invasive Species Lists, 2015. Government Gazette, 493, May 29. (Regulation Gazette No. 38833)

[CR63] South Africa. Department of Environmental Affairs (2015) Feral pigeon invasive species Available via DIALOG. https://www.environment.gov.za/mediarelease/feralpigeon_invasivespecies. Accessed 10 Dec 2015

[CR64] South African Weather Service (2010) Climate of South Africa. WB42 Climate Statistics 0513465. University of Pretoria Proefplaas

[CR65] Tobin ME, Woronecki PP, Dolbeer RA, Bruggers RL (1988). Reflecting tape fails to protect ripening blueberries from bird damage. Wildl Soc Bull.

[CR66] Treves A, Noughton-Treves L (2005) Evaluating lethal control in the management of human-wildlife conflict. In: Woodroffe R, Thiegood S, Rabinowitz A. People and wildlife, conflict or co-existence? Cambridge University Press, New York

[CR67] Wetherbee DK, Coppinger RP, Wentworth BC, Walsh RE (1964) Anti-fecundity effects of Sudan black B and transovarian intravital staining in avian population control. Experimental Station Bulletin, University of Massachusettes

